# Correction: Sizing multimodal suspensions with differential dynamic microscopy

**DOI:** 10.1039/d4sm90050b

**Published:** 2024-03-28

**Authors:** Joe J. Bradley, Vincent A. Martinez, Jochen Arlt, John R. Royer, Wilson C. K. Poon

**Affiliations:** a School of Physics & Astronomy, The University of Edinburgh Peter Guthrie Tait Road Edinburgh EH9 3FD UK w.poon@ed.ac.uk

## Abstract

Correction for ‘Sizing multimodal suspensions with differential dynamic microscopy’ by Joe J. Bradley *et al.*, *Soft Matter*, 2023, **19**, 8179–8192, https://doi.org/10.1039/D3SM00593C.

The authors regret that the data availability statement was not included in the original article. The data availability statement is given below.

 

**Data availability**

Data relevant to this publication is available at https://doi.org/10.7488/ds/3851.

 

The Royal Society of Chemistry apologises for these errors and any consequent inconvenience to authors and readers.

## Supplementary Material

